# Gender-Related Differences in Hospitalization Outcomes of Acute Pancreatitis: A Retrospective Analysis

**DOI:** 10.7759/cureus.86371

**Published:** 2025-06-19

**Authors:** Ahmed Ali Aziz, Muhammad Ali Aziz, Nosheen Omar, Rida Zahid, Muhammad Amir, Rehan Shah, Ijlal Akbar Ali

**Affiliations:** 1 Internal Medicine, Integris Baptist Medical Center, Oklahoma City, USA; 2 Internal Medicine, University of Kentucky, Lexington, USA; 3 Internal Medicine, University of Health Sciences, Lahore, PAK; 4 Internal Medicine, Wah Medical College, Rawalpindi, PAK; 5 Transplant Hepatology, Integris Baptist Medical Center, Oklahoma City, USA; 6 Internal Medicine, Bayonne Medical Center, Bayonne, USA; 7 Section of Digestive Diseases and Nutrition, Department of Medicine, University of Oklahoma Health Sciences Center, Oklahoma City, USA

**Keywords:** acute pancreatitis, alcoholic pancreatitis, biliary pancreatitis, male versus female, nationwide inpatient sample

## Abstract

Background

Acute pancreatitis (AP) is a common gastrointestinal condition that frequently necessitates hospitalization. The aim of our study is to investigate whether gender affects the outcomes of adult patients hospitalized with AP.

Methods

Using the Nationwide Inpatient Sample (NIS) database 2020 and the International Classification of Diseases, Tenth Revision, Clinical Modification (ICD-10-CM) codes, we performed a retrospective study of adult patients admitted with AP. We compared inpatient outcomes of AP between males and females. The primary outcome was all-cause inpatient mortality. Secondary outcomes were length of stay (LOS), total healthcare cost utilization, incidence of acute renal failure (ARF), sepsis, shock, and the need for intensive care unit (ICU) admission. We used STATA Version 16.1 to perform the statistical analyses. Multivariate logistic regression analysis was conducted to assess if gender was an independent predictor for these outcomes and to adjust for any confounders.

Results

A total of 252,595 adult patients were admitted for AP in 2020. The mean age was 50.89 years, and 139,180 (55.1 %) of the patients were males. Female patients had a higher prevalence of chronic kidney disease (9.26% vs 8.47%, P < 0.01), congestive heart failure (6.48% vs 5.69%, P < 0.01), and obesity (23.37% vs 17.39%, P < 0.01). Male patients had a higher prevalence of diabetes mellitus type 2 (27.32% vs 24.68%, P < 0.01), diabetes mellitus type 1 (1.32% vs 1.25%, P = 0.5), liver cirrhosis (1.81% vs 1.75%, P = 0.61), and smoking/tobacco use (39.38% vs 29.28%, P < 0.01). Females had significantly lower likelihood of in-hospital mortality (aOR: 0.64, 95% CI: 0.51-0.82, P < 0.01), ARF (aOR 0.72, 95% CI: 0.68-0.76, P < 0.01), sepsis (aOR: 0.68, 95% CI: 0.63-0.75, P < 0.01), and shock (aOR: 0.74, 95% CI: 0.62-0.89, P < 0.01) than males. There was no statistically significant difference between LOS, total hospitalization charges, and ICU admission between the two genders.

Conclusions

We found that females had significantly improved clinical outcomes, including lower mortality, ARF, sepsis, and shock, as compared to males. There was no statistical difference between the two genders in LOS, total hospitalization charges, and ICU admission. Further prospective studies are needed to accurately understand these differences to guide clinical practice.

## Introduction

Acute pancreatitis (AP) is an acute inflammatory disease of the pancreas. It is one of the very common gastrointestinal diseases that require hospital admission [1]. Common causes of AP include gallstones, alcohol use, autoimmune disorders, and drugs [2]. Alcohol-induced acute pancreatitis (AIP) is more common in males, and gallstone-induced acute pancreatitis (GIP) is more common in females [3,4]. Gender has been known to play a role in the etiology, prevalence, and outcomes of various diseases. Studying gender-based differences in disease outcomes can help provide a greater insight into the disease mechanism and help devise targeted treatment therapy for genders [5]. Over the years, the incidence and prevalence of AP have been increasing, leading to increased hospitalizations of patients with AP [1]. This creates a need to understand this disease and its inpatient hospitalization outcomes better. There are limited studies on the epidemiologic characteristics and outcomes of AP in males and females separately. Hence, we conducted a study using the Nationwide Inpatient Sample (NIS) database to determine gender-specific outcomes in adult patients hospitalized with AP.

## Materials and methods

Data source

We conducted a retrospective analysis using the 2020 NIS database. The NIS, developed by the Healthcare Cost and Utilization Project (HCUP), is the largest publicly available inpatient healthcare database in the United States. It includes data from 48 states, covering over 98% of the U.S. population. Drawn from the State Inpatient Databases, the NIS contains information on approximately 7 million hospital stays annually. It represents a 20% stratified sample of all hospital admissions nationwide, allowing for accurate national estimates of disease prevalence and comorbidities. Each hospital discharge in the sample is assigned a weight, calculated as the total number of discharges from all U.S. acute care hospitals divided by the number of discharges in the 20% sample, making the data nationally representative. When weighted, the NIS reflects around 35 million hospitalizations per year across the country [6]. The database includes detailed information on patient demographics, admission details, discharge diagnoses, length of stay (LOS), and total hospitalization charges. For this study, we utilized diagnostic codes from the International Classification of Diseases, Tenth Revision, Clinical Modification (ICD-10-CM), provided by the World Health Organization (WHO) (https://www.cdc.gov/nchs/icd/icd-10-cm/index.html).

Study population

The ICD-10-CM code “K85” was used to identify all patients with a primary discharge diagnosis of AP. NIS divides patients between the genders: male and female. We divided patients with a primary diagnosis of AP into male patients and female patients. Patients were excluded if they had an elective admission, died on the day of admission, were <18 years old, or were transferred from another hospital. We also excluded hospitalizations with non-positive NIS weights and those missing any patient- or hospital-level characteristics. Because NIS is a publicly available administrative database, we did not require an Institutional Review Board (IRB) approval to conduct this study.

Study variables

Our study variables included age, income quartile, biologic sex, hospital location, primary payer (Medicare, Medicaid, private, self-pay), hospital teaching status, hospital bed size, and race (White, Black, Hispanic, Asian/Pacific Islander, Native American, Other). We used the Charlson comorbidity index (CCI) to assess the burden of comorbidities.

Study outcomes

All-cause in-hospital mortality was the primary outcome. Mean total hospitalization charges, mean LOS, incidence of shock, sepsis, acute renal failure (ARF), and the need for intensive care unit (ICU) admission were secondary outcomes.

Statistical analysis

We conducted our statistical analysis using STATA Version 16.1 (StataCorp., College Station, Texas, USA). This software enables analysis of the NIS database to generate nationally representative results. Categorical variables were compared using the chi-square test, while continuous variables were analyzed using Student's t-test. Univariate analysis was first performed to evaluate the relationship between individual variables and outcomes. Variables with a P-value less than 0.2 were included in the multivariate regression analysis, which was used to estimate outcomes while adjusting for potential confounders. Adjustments were made for factors such as age, insurance status, race, hospital teaching status, hospital bed size, hospital location, and CCI. Logistic regression was applied for binary outcomes, and linear regression was applied for continuous outcomes. A P-value of less than 0.05 was considered statistically significant for all analyses.

## Results

Patient and hospital characteristics

The 2020 NIS database had over 32 million hospital-weighted discharges, of which 252,595 had a principal discharge diagnosis of AP. The mean age was 50.89 years, and 113,415 (44.9%) patients were females. Majority of the patients, 160,751 (63.64%), were white. Most patients had a median income of $1-$51,999 and were admitted to teaching hospitals. Of the patients, 78,506 (31.07%) patients had alcoholic pancreatitis, while 40,895 (16.2%) patients had biliary pancreatitis. The complete patient and hospital characteristics of all patients with a primary diagnosis of AP are presented in Table 1.

**Table 1:** Patient and hospital characteristics of all patients with the primary diagnosis of acute pancreatitis

Variable	N (%)
Total study population	252,595
Female	113,415 (44.9%)
Male	139,180 (55.9%)
Mean age (in years)	50.89
Insurance provider	
Medicare	71,813 (28.43%)
Medicaid	68,983 (27.31%)
Private	84,493 (33.45%)
Self-pay	27,305 (10.81%)
Charlson comorbidity index	
0	93,005 (36.82%)
1	80,325 (31.80%)
2	37,435 (14.82%)
3 or more	41,830 (16.56%)
Median income in patient zip code	
$1-$51,999	81,134 (32.12%)
$52,000-$65,999	69,969 (27.70%)
$66,000-$87,999	58,324 (23.09%)
>$88,000	43,168 (17.09%)
Hospital region	
Northeast	41,931 (16.60%)
Midwest	58,147 (23.02%)
South	101,947 (40.36%)
West	50,570 (20.02%)
Hospital location/teaching status	
Rural	27,937 (11.06%)
Urban Nonteaching	52,969 (20.97%)
Urban Teaching	171,689 (67.97%)
Hospital size	
Small	68,504 (27.12%)
Medium	73,151 (28.96%)
Large	110,940 (43.92%)
Race	
White	160,751 (63.64%)
Black	44,128 (17.47%)
Hispanic	33,139 (13.12%)
Asian or Pacific Islander	5,157 (2.042%)
Native American	2,222 (0.88%)
Other	7,198 (2.85%)
Comorbidity	
Congestive heart failure	15,282 (6.05%)
Liver cirrhosis	4,521 (1.79%)
Diabetes mellitus type 1	3,258 (1.29%)
Diabetes mellitus type 2	66,028 (26.14%)
Chronic kidney disease	22,279 (8.82%)
Obesity	50,696 (20.07%)
Smoker/nicotine dependence	88,004 (34.84%)
Etiology	
Idiopathic	10,306 (4.08%)
Biliary	40,895 (16.19%)
Alcoholic	78,506 (31.08%)
Other	122,888 (48.65%)

**Figure 1:**
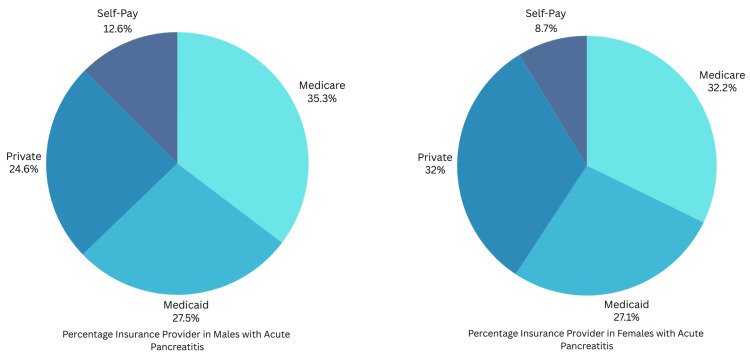
Percentage insurance distribution between males and females with acute pancreatitis

In both males and females with AP, Medicare was the highest insurance provider, with 49,131 (35.3%) and 36,520 (32.2%), respectively. Figure 1 shows the insurance distribution between males and females.

**Figure 2:**
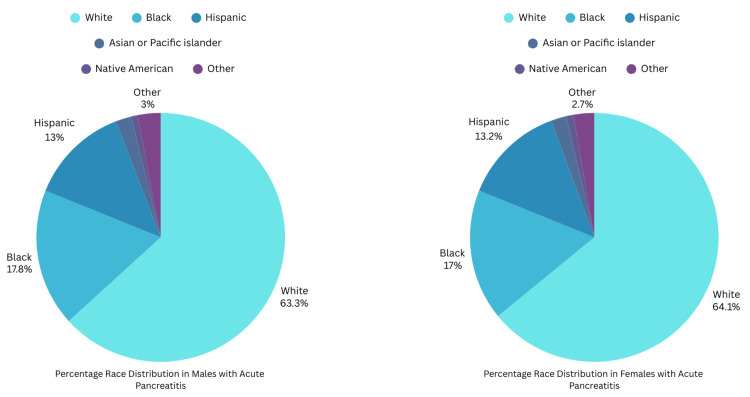
Race distribution in males and females with acute pancreatitis

Caucasian was the most prevalent race in both males and females with AP (Figure 2).

**Figure 3:**
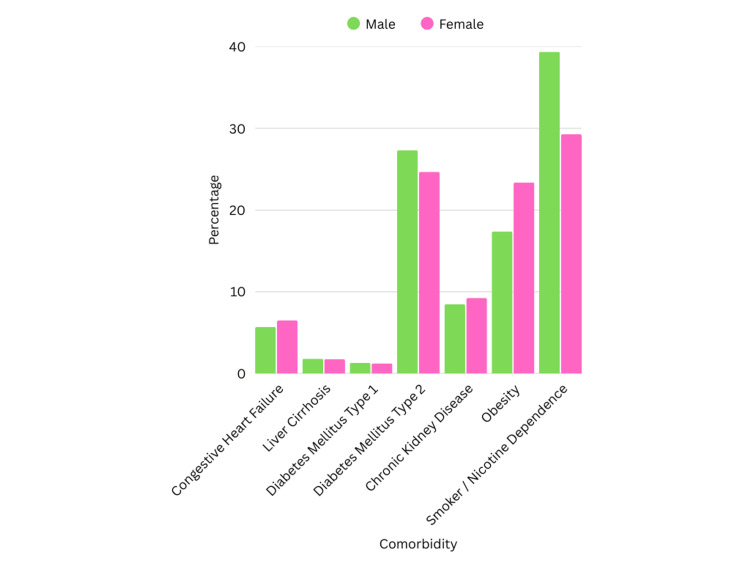
Comorbidities between males and females with acute pancreatitis

Male patients were older than females with a mean age of 52 years, majority were white 88,059 (63.27%). Female patients were younger with a mean age of 49.9 years, and majority were white 72,699 (64.1%). Males had a higher prevalence of diabetes mellitus type 1 (DM1) (1.32% vs 1.25%, P = 0.5), diabetes mellitus type 2 (DM2) (27.32% vs 24.68%, P < 0.01), liver cirrhosis (1.81% vs 1.75%, P = 0.61), and smoking/tobacco use (39.38% vs 29.28%, P < 0.01). Females had a higher prevalence of chronic kidney disease (CKD) (9.26% vs 8.47%, P < 0.01), congestive heart failure (6.48% vs 5.69%, P <0.01), and obesity (23.37% vs 17.39%, P < 0.01) (Figure 3).

Gender-stratified patient and hospital characteristics are presented in Table 2.

**Table 2:** Gender-stratified patient and hospital characteristics of patients admitted with acute pancreatitis Significant *P*-value < 0.05

Variable	Male	Female	P-value
Proportion (%)	139,180 (55.1%)	113,416 (44.9%)	
Mean age in years	52.0	49.9	<0.01 (t-test)
Etiology (%)			
Idiopathic	4,746 (3.41%)	5,569 (4.91%)	<0.01 (chi-square test)
Biliary	16,646 (11.96%)	24,260 (21.39%)	
Alcoholic	54,196 (38.94%)	24,316 (21.44%)	
Other	63,591 (45.69%)	59,271 (52.26%)	
Insurance provider (%)			
Medicare	49,131 (35.3%)	36,520 (32.2%)	<0.01 (chi-square test)
Medicaid	38,303 (27.52%)	30,690 (27.06%)	
Private	34,279 (24.63%)	36,316 (32.02%)	
Self-pay	17,466 (12.55%)	9,890 (8.72%)	
Charlson comorbidity index (%)			
0	49,812 (35.79%)	43,189 (38.08%)	< 0.01 (chi-square test)
1	45,776 (32.89%)	34,569 (30.48%)	
2	20,807 (14.95%)	16,615 (14.65%)	
3 or more	22,784 (16.37%)	19,043 (16.79%)	
Median income in patient zip code (%)			
$1-$51,999	44,523 (31.99%)	36,633 (32.3%)	0.06 (chi-square test)
$52,000-$65,999	38,497 (27.66%)	31,462 (27.74%)	
$66,000-$87,999	31,803 (22.85%)	26,516 (23.38%)	
>$88,000	24,356 (17.5%)	18,805 (16.58%)	
Hospital region (%)			
Northeast	23,354 (16.78%)	18,578 (16.38%)	0.25 (chi-square test)
Midwest	32,359 (23.25%)	25,802 (22.75%)	
South	55,922 (40.18%)	46,035 (40.59%)	
West	27,544 (19.79%)	23,001 (20.28%)	
Hospital location/teaching status (%)			
Rural	15,463 (11.11%)	12,464 (10.99%)	0.31 (chi-square test)
Urban nonteaching	29,506 (21.2%)	23,466 (20.69%)	
Urban teaching	94,210 (67.69%)	77,486 (68.32%)	
Hospital size (%)			
Small	37,689 (27.08%)	30,792 (27.15%)	0.26 (chi-square test)
Medium	40,766 (29.29%)	32,460 (28.62%)	
Large	60,724 (43.63%)	50,164 (44.23%)	
Race (%)			
White	88,059 (63.27%)	72,699 (64.1%)	0.04 (chi-square test)
Black	24,829 (17.84%)	19,315 (17.03%)	
Hispanic	18,107 (13.01%)	15,005 (13.23%)	
Asian or Pacific Islander	2,853 (2.05%)	2,291 (2.02%)	
Native American	1,169 (0.84%)	1,066 (0.94%)	
Other	4,162 (2.99%)	3,040 (2.68%)	
Comorbidity (%)			
Congestive heart failure	7,919 (5.69%)	7,349 (6.48%)	0.00 (chi-square test)
Liver cirrhosis	2,519 (1.81%)	1,984 (1.75%)	0.62 (chi-square test)
Diabetes mellitus type 1	1,837 (1.32%)	1,417 (1.25%)	0.51 (chi-square test)
Diabetes mellitus type 2	38,026 (27.32%)	27,991 (24.68%)	<0.01 (chi-square test)
Chronic kidney disease	11,788 (8.47%)	10,502 (9.26%)	<0.01 (chi-square test)
Obesity	24,203 (17.39%)	26,505 (23.37%)	<0.01 (chi-square test)
Smoker/nicotine dependence	54,808 (39.38%)	33,208 (29.28%)	<0.01 (chi-square test)

Etiology

Of the 252,595 adult patients admitted for AP, 78,506 (31.08%) had AIP, of which 24,316 (21.44%) were females and 54,196 (38.94%) were males. Also, 40,895 (16.19%) of patients had GIP, of which 24,260 (21.39%) were females and 16,646 (11.96%) were males.

Length of hospital stay

We used linear regression to calculate the mean length of hospital stay between males and females with AP. We adjusted for possible confounders including age, Charlson comorbidity disease severity index, race, patients’ insurance status, hospital teaching status, hospital bed size, and hospital location using multivariate linear regression analysis. We found that in all patients admitted with AP, the mean LOS was 4.23 days. It was 4.25 days in males and 4.20 days in females. Female patients with AP had shorter hospital LOS than males (-0.0466 days, 95% CI: -0.13 to 0.38, P = 0.28) after adjusting for confounders using multivariate linear regression analysis. This difference, however, was not statistically significant.

Total hospitalization charges

We used linear regression to calculate the total hospitalization charges between males and females with AP. We adjusted for possible confounders including age, Charlson comorbidity disease severity index, race, patients’ insurance status, hospital teaching status, hospital bed size, and hospital location using multivariate linear regression analysis. We found that for all patients admitted with AP the mean total hospitalization charges were $45,036. The mean total hospitalization charges for males were $44,990 and for females were $45,093. Females had higher total hospitalization charges than males (+ $44.2, 95% CI: -1312.3 to 1400.7, P = 0.94) after adjusting for confounders using multivariate linear regression analysis. This difference, however, was not statistically significant.

Acute renal failure

We used logistic regression analysis to calculate the odds of ARF in males versus females with AP. We adjusted for possible confounders including age, Charlson comorbidity disease severity index, race, patients’ insurance status, hospital teaching status, hospital bed size, and hospital location using multivariate logistic regression analysis. We found that the overall incidence of ARF in all patients admitted with AP was 33,317 (13.19%). Moreover, 20,237 (14.54%) males with AP developed ARF and 13,088 (11.54%) females with AP developed ARF. Females with AP had 28% lower odds of developing ARF as compared to males (aOR 0.72, 95% CI: 0.68-0.76, P < 0.01) after adjusting for confounders using multivariate logistic regression analysis. This difference was statistically significant.

Sepsis

We used logistic regression analysis to calculate the odds of sepsis in males versus females with AP. We adjusted for possible confounders including age, Charlson comorbidity disease severity index, race, patients’ insurance status, hospital teaching status, hospital bed size, and hospital location using multivariate logistic regression analysis. Our results showed that 14,246 (5.64%) patients with AP developed sepsis, and 9,088 (6.53%) males and 5,160 (4.55%) females with AP developed sepsis. Females with AP had 32% lesser odds of developing sepsis as compared to males with AP (aOR: 0.68, 95% CI: 0.63-0.75, P < 0.01) after adjusting for confounders using multivariate logistic regression analysis. This difference was statistically significant.

Shock

We used logistic regression analysis to calculate the odds of shock in males versus females with AP. We adjusted for possible confounders including age, Charlson comorbidity disease severity index, race, patients’ insurance status, hospital teaching status, hospital bed size, and hospital location using multivariate logistic regression analysis. We found that 2,778 (1.1%) patients with AP developed shock, and 1,740 (1.25%) males and 1,043 (0.92%) females with AP developed shock. Females with AP had 26% lesser odds of developing shock than males with AP (aOR: 0.74, 95% CI: 0.62-0.89, P = 0.001) after adjusting for confounders using multivariate logistic regression analysis. This difference was statistically significant.

ICU admission

We used logistic regression analysis to calculate the odds of ICU admission in males versus females with AP. We adjusted for possible confounders including age, Charlson comorbidity disease severity index, race, patients’ insurance status, hospital teaching status, hospital bed size, and hospital location using multivariate logistic regression analysis. We found that 8,714 (3.45%) patients with AP required ICU admission, and 4,746 (3.41%) males and 3,958 (3.49%) females with AP required ICU admission. Females with AP had 4% lesser odds of requiring ICU admission than males (aOR 0.96, 95% CI: 0.86 -1.07, P = 0.49) after adjusting for confounders using multivariate logistic regression analysis. This difference, however, was not statistically significant.

In-hospital mortality

We used logistic regression analysis to calculate the odds of in-hospital mortality in males versus females with AP. We adjusted for possible confounders including age, Charlson comorbidity disease severity index, race, patients’ insurance status, hospital teaching status, hospital bed size, and hospital location using multivariate logistic regression analysis. Our results showed that the total all-cause in-hospital mortality for all patients with AP was 1,541 (0.61%), and 974 (0.70%) males and 567 (0.50%) females with AP died while hospitalized. As compared to males, females with AP had 36% lesser odds of in-hospital mortality (aOR: 0.64, 95% CI: 0.51-0.82, P < 0.01) after adjusting for confounders using multivariate logistic regression analysis. This difference was statistically significant.

## Discussion

AP is a common gastrointestinal condition that frequently necessitates hospitalization. Due to current lifestyle, such as high-fat diets, rising obesity rates, increased alcohol intake, and a growing prevalence of gallstone disease, the national incidence of AP is on the rise [7,8]. Gender has been shown to affect the outcomes of various diseases. The impact of gender on outcomes of AP remains less studied. Our study is one of the rare large-scale and nationwide cohort studies to evaluate outcomes of AP in males and females.

We found that females with AP had shorter hospital LOS than males. We believe that there are multiple reasons for this. First, our results show that females had a relatively higher prevalence of GIP and a relatively lower prevalence of AIP as compared to males, who had a higher prevalence of AIP. GIP tends to have a more predictable and often less severe course than AIP, which may be associated with more complications and longer recovery times as compared to GIP, contributing to the longer hospital LOS in males [9]. AIP is also usually associated with systemic inflammatory response syndrome (SIRS), requiring a longer time for management, treatment, and resolution [10]. Females also have a more healthcare-seeking behavior. Their tendency to seek care early leads to earlier presentation to the hospital, earlier intervention, and hence shorter stays [11]. Our results also showed that the male population with AP was relatively older than females, and the prevalence of comorbid conditions such as liver cirrhosis, DM1, DM2, and tobacco use disorder was higher in males, making the male population relatively sicker than the female population with AP and contributing to longer LOS in males. We believe that all of these reasons contributed to a longer LOS in males with AP than females.

We found that females had lower odds of developing ARF than males. This is likely because males had a relatively higher prevalence of AIP, which is generally more severe and is associated with SIRS [9,10]. People with alcohol intoxication also have severe ongoing nausea, vomiting, and decreased appetite, which all contribute to dehydration and pre-renal ARF; hence, we noted higher odds of ARF in males as compared to females [12].

We found that females with AP had lower odds of developing sepsis than males. We believe that there are several reasons for males having higher odds of developing sepsis. Male population was relatively older and had a higher prevalence of comorbidities such as liver cirrhosis, DM1, DM2, and tobacco use disorder, predisposing them to develop sepsis. Males had a relatively higher prevalence of AIP, while females had a relatively higher prevalence of GIP. GIP tends to be less severe than AIP [9]. AIP is also associated with SIRS, and alcohol consumption may compromise patients’ natural immune system and make them prone to developing sepsis [10,13]. The healthcare-seeking behavior of females leads to early presentation, early intervention, and lower odds of developing severe disease and sepsis [11]. The estrogen hormone prevalent in females has also been shown to enhance bacterial clearance [14], while testosterone might suppress certain immune functions [15]. This might also be a part of the explanation why females have lower odds of developing sepsis in AP.

We found that females had lower odds of developing shock and ICU admission as compared to males. We believe that this is due to lower odds of ARF and sepsis in females; the relatively higher prevalence of GIP than AIP in females and the early healthcare-seeking nature of females are responsible for lower odds of shock and ICU admission in females. Due to lower odds of requiring ICU admission and shock in females, we also noted that the overall inpatient mortality in females was lower as compared to males with AP.

Our study has several limitations. It relies on the NIS database, which uses ICD-10 codes for diagnosis. There is a possibility that some patients were inaccurately coded as having AP, potentially introducing bias into the dataset. Nevertheless, the large sample size likely minimizes the impact of such errors on our overall findings. Since the NIS does not support randomization, we employed a multivariate regression model to control for confounding variables, including a range of patient- and hospital-level factors. As a retrospective analysis, our study can identify associations but cannot determine causation. Additionally, the NIS database does not provide information on the severity of AP, preventing us from analyzing outcomes based on disease severity.

Our study offers several notable strengths. It benefits from a large sample size and broad geographic representation, drawing on data from the NIS database. Because the NIS is a stratified, nationally representative sample of the U.S. population, our findings are more generalizable to the national level. This enhances the statistical power of the study and reduces the risk of selection bias. In contrast to many epidemiologic studies that are limited to data from a single state or a few centers, thus limiting generalizability, our study provides a more comprehensive perspective. Additionally, we strengthened our analysis by employing multivariate regression to adjust for a wide range of potential confounders, including age, race, gender, median income quartile, CCI, and various hospital characteristics.

## Conclusions

Gender-related differences in the inpatient outcomes of patients hospitalized with AP is a less studied topic. Our study highlights several important findings for clinicians to keep in mind in their daily practice. Males with AP have high mortality and morbidity, and early recognition and treatment of AIP in males can save hospital LOS, total hospitalization charges, morbidity, and mortality. AP is more severe in males, as evidenced by the increased mortality, ARF, sepsis, and shock, when compared to females and thus warrants close monitoring and early and aggressive treatment. Our study is a useful study to help stratify hospitalized patients with AP and allocate healthcare resources appropriately.
